# Psychological Aspects of Congenital Hypogonadotropic Hypogonadism

**DOI:** 10.3389/fendo.2019.00353

**Published:** 2019-07-05

**Authors:** Andrew A. Dwyer, Neil Smith, Richard Quinton

**Affiliations:** ^1^William F. Connell School of Nursing, Boston College, Boston, MA, United States; ^2^Reproductive Endocrine Unit, Massachusetts General Hospital, Boston, MA, United States; ^3^International Patient Support Group for Hypogonadotropic Hypogonadism (HYPOHH), London, United Kingdom; ^4^Newcastle-upon-Tyne Hospitals Foundation NHS Trust (Royal Victoria Infirmary) and Institute of Genetic Medicine, University of Newcastle-upon-Tyne, Newcastle-upon-Tyne, United Kingdom

**Keywords:** coping, hypogonadotropic hypogonadism, kallmann syndrome, patient activation, patient centered care, patient experience, transitional care

## Abstract

Congenital hypogonadotropic hypogonadism/Kallmann syndrome (CHH/KS) is a rare, treatable form of infertility. Like other rare disease patients, individuals with CHH/KS frequently experience feelings of isolation, shame, and alienation. Unlike many rare diseases, CHH/KS is not life threatening and effective treatments are available. Nevertheless, it remains a profoundly life-altering condition with psychosocial distress on a par with untreatable or life-limiting disease. Patients with CHH/KS frequently express lasting adverse psychological, emotional, social, and psychosexual effects resulting from disrupted puberty. They also frequently experience a “diagnostic odyssey,” characterized by distressing and convoluted medical referral pathways, lack-of-information, misinformation, and sometimes-incorrect diagnoses. Unnecessary delays in diagnosis and treatment-initiation can significantly contribute to poor body image and self-esteem. Such experiences can erode confidence and trust in medical professionals as well as undermine long-term adherence to treatment–with negative sequelae on health and wellbeing. This review provides a summary of the psychological aspects of CHH/KS and outlines an approach to comprehensive care that spans medical management as well as appropriate attention, care and referrals to peer-to-peer support and mental health services to ameliorate the psychological aspects of CHH/KS.

## Introduction

Congenital hypogonadotropic hypogonadism (CHH) is a rare genetic endocrine disorder causes by the insufficient secretion or action of gonadotropin-releasing hormone (GnRH). Biochemically it is defined by very low sex steroid levels (testosterone, estradiol) in the setting of low or inappropriately normal serum levels of gonadotropins (luteinizing hormone, follicle stimulating hormone) (1). Clinically, the condition manifests as absent or incomplete puberty with infertility. Thus, without exogenous hormonal therapy, individuals remain in a state of arrested pubertal development. Individuals typically exhibit some scant signs of androgenization (i.e., scant/Tanner II axillary and pubic hair) arising from secretion of weak androgens by the adrenal glands. In males, the absence of normal sex steroid levels is evidenced by lack of virilization, i.e., poor muscle development, gynoid habitus, sparse body hair, high-pitched voice, and undeveloped genitalia. In females, there are little to no secondary sexual characteristics (i.e., breast development, pubic hair) and absent menses (amenorrhea).

Additionally, clinical presentation may be accompanied by a variety of highly variable non-reproductive phenotypes (Box 1) (1, 2). Notably, when patients exhibit a diminished/altered sense of smell (hyposmia/anosmia) it is termed Kallmann syndrome (KS). Associated phenotypes occur at highly variable rates. Thus, patients present on a spectrum ranging from relatively milder forms (e.g., CHH with normal sense of smell and partial puberty) to more severe, syndromic forms of CHH (e.g., Kallmann syndrome with complete absence of puberty, unilateral renal agenesis and cleft lip/palate) (3).

Box 1 **Signs of CHH/KS and associated phenotypes.**

**Table:** 

**Hallmark signs of absent/incomplete puberty**	
Males	Females
• High-pitched voice	Absent/limited breast development
• No beard development	
• Lack of muscle mass	• Undeveloped feminine figure
• Scant body/pubic hair	• Scant pubic hair
• Underdeveloped genitals	• Primary amenorrhea
**ASSOCIATED PHENOTYPES**	
**Reproductive**	
• Maldescended testes (cryptorchidism)	
• Micropenis	
**Sensory and Neurologic**	
• Defective sense of smell (hyposmia/anosmia)	
• Sensorineural hearing loss	
• Ocular and oculomotor defects (coloboma, micropthalmia)	
• Mirror movement (synkinesia)	
• Ataxia (Gordon-Holmes syndrome)	
**Musculo-Skeletal**	
• Eunuchoidal proportions	
• Scoliosis, osteopenia/osteoporosis	
• High arched palate	
• Cleft lip/palate	
• Dental agenesis	
• Digit anomalies (syndactyly, clinodactyly, split hand-foot)	
**Dermatologic**	
• Pigmentation defects (achromic patches)	
• Ichthyosis	
**Internal Organs**	
• Renal agenesis (unilateral)	
• Heart defects	

## The Challenge of Diagnosis

In parallel to the clinical heterogeneity of CHH, the molecular basis is likewise diverse and complex (4). Inheritance patterns include X-linked, autosomal recessive, autosomal dominant, as well as digenic and oligogenic forms (5). Since the early 1990's, more than 30 genetic loci have been identified to underlie CHH/KS. Significant advances have been made in understanding the molecular basis of CHH/KS yet the known genes only account for ~50% of cases (1). As such, genetic testing may be informative in helping to confirm a diagnosis in less than half of cases, with the mainstay of diagnosis remains based on clinical ascertainment and biochemical measurement of serum hormones.

Importantly, CHH can be a difficult diagnosis to make. The hallmark signs of CHH include failure to initiate spontaneous puberty or inability to maintain progressive pubertal development. In the general population, pubertal onset is highly variable. One may view a photograph of a middle school class picture and quite easily see that some students have yet to begin puberty (e.g., short stature and Tanner I) while other classmates have begun or are well into puberty (e.g., growth spurt, acne, facial hair development in boys and breast development in girls). Indeed, delayed puberty statistically defined by the bell-shaped curve of puberty (6). Constitutional delay of growth and puberty (CDGP) occurs in 2.5% of the population and represents those individuals at the far tail of the distribution who will undergo spontaneous puberty—yet will do so significantly later than their peers.

Currently, there is no gold-standard test to differentiate CDGP from CHH. A number of serum biomarkers have been identified and dynamic tests have been developed and evaluated. To date, all these approaches lack appropriate sensitivity and specificity to accurately tease apart delayed spontaneous puberty and abiding absent puberty (7). In some cases, clinical “red flags” may point to a diagnosis (1, 8–10) (Box 2). Unfortunately, such clinical signs often go unrecognized (or their significance is not appreciated) and a “watchful waiting” approach is taken (11). Making a diagnosis is complicated and difficult because CHH/KS is a diagnosis of exclusion and other potential causes (i.e., functional, iatrogenic and tumors) must be ruled-out (1, 12).

Box 2 **Red flags pointing to CHH/KS diagnosis.**

**Table:** 

**Positive family history—including offspring of CHH patients secondary to fertility-inducing treatment**
**Signs of absent mini puberty (first 6-months of life)**
° Maldescended testes (unilateral or bilateral cryptorchidism)
° Micropenis
° No erections noted during diaper changes
Absent sense of smell (anosmia)—typically not evident until age 6–8 years
Presence of midline or skeletal defects
° Cleft lip and/or palate
° Syndactyly (webbing) or other anomaly of digits

**Figure 1:**
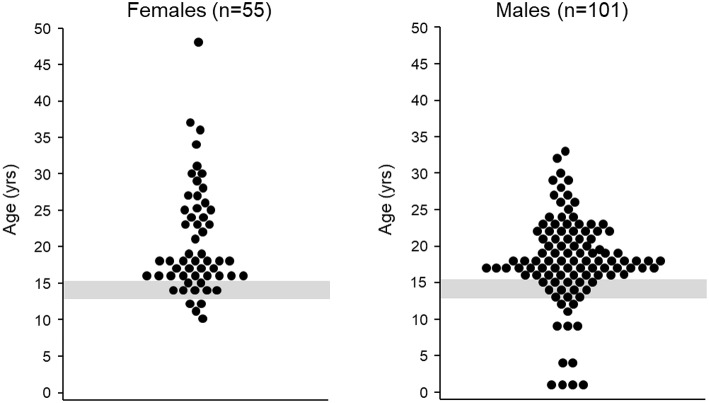
Age at diagnosis. **Left** age at CHH/KS diagnosis for female patients (*n* = 55), median age at diagnosis = 18 years (mean: 21 ± 7 years). The gray shaded region depicts the mean age of menarche plus two standard deviations (15). Figure adapted from Dzemaili et al. (16). **Right** age at CHH/KS diagnosis for male patients (*n* = 101), median age at diagnosis = 18 years (mean: 18 ± 6 years, The gray shaded region depicts mean age of genital stage 3 plus two standard deviations (17). Figure adapted from Dwyer et al. (18).

Although a detailed three-generation family pedigree provides important genetic insights to a case, it may not always be informative for CHH/KS diagnostics. Studies demonstrate that pedigrees of patients with CHH/KS are enriched with family members with a history of delayed puberty (13, 14). Thus, clinicians may incorrectly assume that the individual is genetically programmed for late puberty. This can result in a “watchful waiting” approach and a missed opportunity for earlier diagnosis resulting from a more active investigation. Being labeled a “late developer” or “late bloomer” may be difficult for teens to accept and they may not feel healthcare professionals are taking them seriously. Such feelings may further inhibit patients discussing their puberty and seeking help for what may be a highly sensitive and embarrassing condition. Guidelines and review articles on delayed puberty are invariably directed at the evaluation and treatment of individuals for whom the cause of pubertal delay may not be initially obvious. However, a “watchful waiting” lacks a logical basis for those individuals with “red flag” features (Box 2) indicating high pre-test probability of CHH/KS. In such cases, sex hormone replacement therapy should not be delayed beyond median age of pubertal onset. Cumulatively, all these factors often contribute to a late diagnosis (Figure 1).

Moreover, patients with rare diseases often experience a “diagnostic odyssey,” including incorrect diagnoses, incomplete information, delays in finding expert care and accurate diagnosis, and misleading or frankly incorrect advice along the way from non-specialists. Such experiences can significantly erode patient confidence in healthcare providers and health systems and affect quality of life (19). For patients with CHH/KS, there is all too often an unacceptable and inexplicable delay between the age of presentation (e.g., at 3-months of age with bilateral cryptorchidism and micropenis) and the age at diagnosis (e.g., at age 50 years with spontaneous vertebral fracture).

## Misconceptions Regarding Treatment

Unlike many rare diseases, there are simple, effective, affordable treatments available for CHH/KS. Exogenous sex steroids can safely and effectively induce development of secondary sexual characteristics in males and females [reviewed in: (1, 20, 21)]. Similarly, exogenous gonadotropins (human chorionic gonadotropin ± recombinant FSH or pulsatile GnRH) can make fertility possible in roughly 75–80% of male patients [reviewed in: (1, 22–25)]. For female patients, ovulation induction can be achieved using exogenous gonadotropins (FSH for follicle development followed by hCG to induce ovulation) yet pulsatile GnRH is the preferred treatment for fertility induction due to decreased risk for multiples (26). If gonadotropins are used for ovulation induction, the risk of multiples can be mitigated by careful serial ultrasound monitoring to ensure that only one single dominant follicle is ovulated following hCG administration. Unfortunately, many patients are incorrectly labeled as “sterile” and remain unaware that fertility is likely possible with specialized regimens. For decades, using low dose testosterone esters (in males) and low dose estradiol (in females) has been the standard treatment to induce secondary sexual characteristics. However, to date, there is no standardized regimen to guide the induction of secondary sexual characteristics, particularly in older adolescents or those of adult age.

An important principle is starting with low dose replacement and gradually escalating dosage in order to maximize growth and attain normal breast contour in females. Importantly, females started directly on full-dose estrogen and progesterone (i.e., hormone replacement therapy, combined oral contraceptive pills) typically achieve markedly suboptimal breast development (1). It remains unclear if optimal breast development results from the progressive, incremental dose increase in estrogen, or an indirect effect of delaying the introduction of progesterone as long as possible. The classical approach is to introduce progestin when girls begin to experience significant vaginal spotting/bleeding on unopposed estrogen. Perhaps a more logical approach might be to monitor endometrial thickness by ultrasound to guide the adjustment of estrogen dose, and thereby prolong the introduction of progesterone until breast development is deemed appropriate. This point is relevant because breast size and appearance can be a source of significant anxiety and may impair body image for many eugonadal women–let alone those women with CHH/KS. Once cyclical estradiol and progesterone therapy has been initiated, some women will incorrectly presume that their regular withdrawal bleeds will be associated with natural ovulation. Hence, anticipatory guidance regarding fertility is important. Additionally, monthly menses may be bothersome to many women with CHH/KS—and potentially undermine adherence to avoid menses. Patients should be informed they can safely have a few withdrawal bleeds per year–rather than menses being a monthly event.

In males, testosterone therapy will induce secondary sexual characteristics, but will not stimulate testicular growth (either gonadotropin therapy or pulsatile GnRH are required for this). Patients are typically not informed of this fact. Accordingly, male patients often have incorrect assumptions that testosterone will induce puberty and normal appearing testes. Lack of appropriate anticipatory guidance and patient education can result in frustration and may erode a therapeutic relationship between the patient and provider—and undermine adherence. Further contributing to frustration and loss-of trust, reviews and guidelines for pubertal-induction emphasize starting with low-dose treatment aiming to complete pubertal maturation over 2–3 years “in line with peer group.” For many patients, this may seem agonizingly slow. For patients diagnosed at an adult-age treatment regimen is both safe and appropriate (27, 28). Once normal sex steroid levels in the serum have been attained, lifelong treatment is required with at least annual monitoring.

Recent studies have revealed that despite the presumed availability of safe and effective treatment, there are major gaps in both anticipatory guidance when initiating treatment as well as significant challenges for long-term adherence to treatment (16, 29, 30). The majority of patients with CHH/KS are diagnosed late (Figure 1) and thus, their physical appearance is much younger than their chronological age. Patients have a strong desire to “catch up” in pubertal development. Patients' desire to more closely resemble peers creates a temporal conflict with the “slow and low” approach to dose increase. Patients may become frustrated without appropriate anticipatory guidance on when changes can be expected. Feelings of dissatisfaction may undermine compliance with treatment (see Targets for Improving Care). CHH/KS is a chronic condition and long-term medication adherence is required for sexual function, bone health, preventing potential metabolic disease and overall well-being (1).

Like many chronic diseases, more than half of patients with CHH/KS struggle with adherence and 48% of women and 38% of men have treatment gaps of more than 1 year (16, 29). Major drivers of adherence include patient beliefs and concerns (31). While patients may be able to find clinicians who are knowledgeable about CHH/KS, evidence suggests that the understanding of the emotional and psychological aspects of care are underappreciated and neglected. A quote from a 1964 article reflects this perspective: “There is a tendency among physicians to assume that a corrigible pathological condition ought to be corrected and that the emotional well-being of the patient will improve concomitantly with his physical condition” (32). The quote was published more than 50 years ago, yet recent data suggest that this view persists. Sixty seven percent of patients believe their provider understands the medical aspects of CHH/KS. However, significantly fewer patients (38%, *p* < 0.001) perceive their provider as understanding the emotional impact of living with CHH/KS (30).

## Challenges Faced by Patients

Rare genetic diseases are often associated with psychological burden and negative emotional and psychosocial effects (33). Some have put forth the notion that challenges and inequities faced by rare disease patients put them in the realm of health disparities (34). Patients with CHH/KS may experience physical, cognitive and psychosocial consequences (Box 3). The lack of sex steroids due to CHH/KS can affect patients physically and cognitively. Physically, sex steroids are critical for bone health—both in formation and maintaining bone density. Because patients with CHH are hypogonadal without treatment, periods without treatment put them at increased risk for compromised bone health (35, 36). Indeed, a Finnish study of 26 patients with CHH found that long periods of non-adherence to treatment was associated with worse bone density (37). Thus, consistent long-term adherence is essential for mitigating the risk for osteopenia and osteoporosis.

In terms of cognition, sex steroids are known to have activational and organizational effects on the brain and neural circuits. Research findings indicate that periods of rising circulating sex steroids (i.e., during the first 6-months of life in the so-called “mini puberty” and during puberty) are important developmental windows in which testosterone and estradiol have sex-specific effects on brain and behavioral development (38). While cognitive impairment is not a hallmark of CHH/KS and most patients have normal IQ, there are data indicating lasting effects on spatial abilities. A study comparing spatial abilities of men with CHH and acquired HH found that deficits observed in patients with CHH were not ameliorated by testosterone treatment. These observations suggest that androgens exert a permanent organizing influence on the brain (39). More recently a study of 34 Lithuanian CHH males at diagnosis (prior to sex steroid treatment) identified significantly lower executive function, attention, visual scanning and psychomotor speed compared to age-matched healthy controls (40). Notably, after 2 years of treatment, scored in these domains were improved—yet without significant changes in either emotional state of quality of life (41).

Approximately half to two-thirds of patients with CHH have diminished/absent sense of smell (i.e., Kallmann syndrome) (1, 42). Patients with defective olfactory function may be prone to eating food/drinks that have spoiled and often have concerns about not being able to detect body odor- contributing to feelings of self-consciousness and insecurity in social situations. Similarly, studies of patients with isolated anosmia reveal associations with increased social insecurity and depressive symptoms (43). One's olfactory acuity is indiscernible to others but absent pubertal development and looking younger than one's age is outwardly evident. Indeed, the disparity between chronological age and appearance can pose a significant barrier for dating and intimate relationships. Puberty is a biologic process that includes physiologic, psychosocial, and emotional changes and adolescence is a period of developing self-concept. Thus, disruption of puberty can carry a psychological burden (6).

Studies in late maturing 14–16 year-old boys identify body image concerns, low self-esteem, social isolation and experiences of teasing and bullying (44, 45)—common risks for depression in adolescents (46). For patients with CHH/KS, experiences are strikingly similar, if not magnified. Survey data indicate 56% of females and 72% of males experience teasing and victimization related to their condition. Body image concerns (e.g., body shame) are reported in 93% of males and 80% of females with CHH/KS. Supplementing these quantitative data, focus groups discussions reveal that concerns about low self-esteem, shame and social isolation are pervasive (16, 18, 30). These experiences often have lasting effects and perhaps not surprisingly, significant impact of intimate relationships and psychosexual development. Prior studies of late maturing boys have revealed they are more dissatisfied with their body image and less sexually active compared to peers who underwent normal pubertal timing (47). Studies in patients with CHH/KS corroborate these findings.

There are anecdotal reports in the literature mentioning low self-esteem and poor body image among patients with CHH/KS (32, 48, 49). Some have posited that pubertal failure and underdeveloped genitalia (i.e., prepubertal testicular size and small penis—both secondary to absent mini puberty) may pose barriers to engaging in sexual activity (50). Online patient discussions are filled with stories highlighting concerns about genital development conveying the impact such concerns have on seeking/initiating intimate sexual relationships. Studies examining quality of life and sexuality in CHH have been sparse with only a few small, anecdotal reports (32, 48, 49). More recently, this topic has gained increased attention. Aydogan et al. reported on 39 Turkish boys with CHH initiating testosterone treatment. Prior to treatment, the young men exhibited increased anxiety, depression, and worse quality of life (using the SF-36) compared to age-matched controls (51). Notably, 6-months of testosterone therapy improved physical function and vitality yet increased anxiety and significant emotional difficulties persisted as they adapted to life as a sexual adult (51). Longer duration of treatment up to 2 years in a cohort of 19 Lithuanian boys also failed to show significant improvements in emotional state and quality of life (40, 41). A Finish study found decreased quality of life in 30 males with CHH who exhibited high levels of distress and depression (52). In 2014, Shiraishi et al. studied six patients undergoing fertility-inducing gonadotropin treatment over 2 years (53). In contrast to testosterone replacement (51), gonadotropin therapy stimulates testicular growth. The investigators observed significant improvement in SF-36 measures including amelioration of the emotional difficulties not seen with testosterone treatment. The authors posited that the genital development might have had a role in improving the emotional and body image concerns of the six patients.

Subsequently, Dwyer et al. reported on the largest cohorts studies to date (101 males, 55 females) providing robust evidence of the psychosexual impact of CHH/KS pervasive (16, 18). Patients found intimate relationships as “very difficult” (68% of males, 59% of females). Females were more likely to have ever been sexually active (89%) compared to male counterparts (74%). Notably, more than a quarter of men with CHH have never been sexually active. This is five-times the rate observed in similarly aged men drawn from a population-based sample (26% vs. 5.4%, *p* < 0.001). In parallel, qualitative focus groups explored the impact of disrupted sexual maturation on psychosexual development and intimate relationships in detail. Patients reported feeling isolated and “left behind” as peers advanced through puberty and started dating and taking on roles that are more adult. Fear and anxiety about being exposed was common and many tried to hide their lack of sexual development and sometimes avoided social interactions—creating a reinforcing and cyclic pattern that lasted well into adulthood (see Targets for Improving Care) (18). Similarly, a Finnish study demonstrated that despite long-term treatment, men with absent mini-puberty (i.e., cryptorchidism with/without micropenis) had the lowest scores on dimensions of sexual activity (52). These data support the notion of persisting body shame and low self-esteem despite long-term treatment with a lasting impact on psychosexual functioning.

For patients with CHH/KS, the pervasive negative illness perceptions provide insights into the burden many patients face (16, 29). Notably, clinicians often underappreciate the psychosocial impact of CHH/KS. Recent research indicates that 34% of patients with CHH/KS exhibit moderate-severe symptoms of depression (16, 29)—yet barely one quarter of patients report ever having a provider discuss psychological support or services. Patients with CHH/KS face a number of challenges that range from feelings of isolation and alienation related to living with a rare disease, possible physical and cognitive issues as well as a constellation of emotional, psychological, and psychosexual problems. It is worthwhile to note that just as the clinical presentation and genetics of CHH/KS are heterogeneous, so too are the coping responses of patients. Some patients struggle with some or many of these challenges yet others effectively cope with these difficulties. The following section outlines areas for improving the care of CHH/KS and avenues for supporting patient empowerment and more patient-centered approaches to care.

## Targets for Improving Care

**Figure 2:**
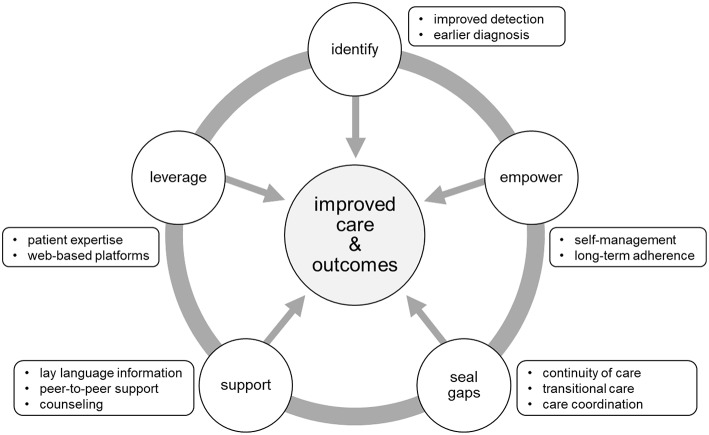
Targets for improving care and outcomes. Five main targets were identified from the literature and feedback from the patient community. Opportunities to improve care and outcomes include: (1) better detection and earlier diagnosis, (2) activating and empowering patients for enhanced chronic disease self-management, (3) promoting continuity of care through care coordination and structured transition from pediatric to adult-oriented care, (4) providing information, enhanced mental health services and access to peer-to-peer support, (5) leveraging technology to extend the reach of care to geographically dispersed patients.

As stated previously, CHH/KS is a difficult diagnosis to make. A major challenge for clinicians and patients alike is the problem of late diagnosis. Healthcare professionals can be frustrated by the genetic heterogeneity of CHH/KS as well as the current lack of plasma/serum biomarkers or sensitive and specific dynamic test to differentiate CDGP and CHH/KS. For patients, the psychosocial ramifications of late diagnosis can have lasting effects. Thus, one of the main targets for improving care and outcomes relates directly to enhanced detection and earlier diagnosis (Figure 2). It is critical to raise clinician awareness of red flags (Box 2). Defective sense of smell (anosmia) is a strong clue for making a KS diagnosis. In male infants, signs of absent mini puberty (e.g., cryptorchidism with/without micropenis) during the neonatal window (first 6-months of life) represent the earliest opportunity to make a diagnosis.

Other opportunities for improving the care and outcomes for patients relate to chronic disease management. Patients with CHH/KS must be activated and empowered for self-care. Indeed, effective, long-term adherence to hormonal treatments are critical for mitigating metabolic risks (e.g., metabolic syndrome, type 2 diabetes) (54) and maintaining bone health (37), sexual function, and well-being. Much has been written on the topic of adherence and the subject is multifaceted and complex yet adopting a patient-centered approach to care is associated with better adherence. Briefly, the Picker Principles of Patient-Centered Care (55) are integral parts of providing high-quality healthcare and include: (1) respect for patients' values, preferences and expressed needs, (2) coordination and integration of care, (3) information, communication and education, (4) physical comfort, (5) emotional support and alleviation of fear and anxiety, (6) involvement of family and friends, (7) continuity and transition, and (8) access to care. Thus, effective patient-provider communication, therapeutic education (including anticipatory guidance) and shared decision-making are key elements for supporting adherence and patient self-management. Patients with CHH/KS often have long gaps in care (16, 30, 37). As with other chronic health conditions, continuity of care is an important factor in reducing complications and improving outcomes. Thus, co-ordinated care and structured transition programs to effectively move young adults form pediatric to adult-oriented care are critical for closing gaps in care (56, 57).

Patients frequently express the desire for more information (16, 30) (Boxes 3, 4). Importantly, healthcare professionals and patients have complementary knowledge and expertise. Providers understand the genetics, pathophysiology and treatment while patients understand what it is like to live with a rare disease. This presents opportunities for co-creating solutions—as recently demonstrated in a project pairing expert clinicians and patients to create patient educational materials in lay language (and translated into 20 languages) (58). Moreover, peer-to-peer support is a means to pierce the veil of isolation many patients feel and offer opportunities for patients to connect and crowdsource solutions (30). There is a need to raise provider sensitivity to the psychosocial aspects of CHH/KS to increase screening for symptoms of anxiety and depression. Further, many patients may benefit from mental health services to address self-esteem and psychosexual issues.

Box 3 Patient identified unmet needs^*^.

**Table:** 

**Knowledge of the CHH/KS**
Patients often have limited understanding of the:
– Clinical difficulty in making a diagnosis
– Possibility of early (neonatal) identification
– Range and severity of signs and symptoms
– Symptoms that may or may not be associated
(e.g., fatigue, cognition/learning/attention problems—including autism spectrum).
**Access to Expert Care**
Patients frequently have difficulty:
– Finding clinicians with experience in diagnosing CHH/KS
– Locating endocrinologists who know how to treat CHH/KS (including fertility-inducing regimens)
**Genetic Testing**
Patients have a poor understanding of:
– The complex genetics of CHH/KS
– What can and can not be achieved through genetic testing (i.e., treatment and potential risk of passing CHH/KS to offspring)
– How to communicate possible risk to family members
**Treatment and Care**
Patients are often unaware of:
– Types of physical/emotional changes that occur with treatment initiation (including timing of changes and what will/will not occur on treatment)
– Necessity of long-term treatment
– Consequences of poor adherence on health and wellbeing
– Treatment options
(including dosage and timing intervals for testosterone injections)
**Psychological and Psychosocial Consequences**
Patients may not perceive:
– The importance of identifying and treating psychological issues related to CHH/KS (including those patients diagnosed early and those who are married with children)
– The life-changing opportunities available through peer-to-peer support
(including body image concerns, self-esteem and sexuality)

* *common concerns and issues raised by patients derived from online peer discussions (personal communication–N. Smith)*.

Box 4 **Resources and links for patients.**

**Table:** 

Medical information and finding experts
https://rarediseases.info.nih.gov/diseases/10771/kallmann-syndrome
https://www.orpha.net/consor/cgi-bin/OC_Exp.php?Lng=GB&Expert=478
https://www.chuv.ch/en/hhn/hhn-home/
**Lay Information**
https://www.youtube.com/watch?v=yKfThHq9Vjs
https://globalgenes.org/raredaily/the-24-year-old-late-bloomer-kallmann-syndrome/
https://en.wikipedia.org/wiki/Kallmann_syndrome
https://www.chuv.ch/en/hhn/hhn-home/
**Patient Perspectives and Peer-to-Peer Support**
Facebook:
– Kallmann Syndrome Links and Help (OPEN group)
– Kallmann Syndromers (CLOSED group)
– Kallmann Syndrome & Hypogonadotropic Hypogonadism (SECRET group)
https://www.news-medical.net/health/Kallmann-Syndrome.aspx
https://www.rareconnect.org/en/community/kallmann-syndrome
https://www.youtube.com/watch?v=eitQYgCqA-0
**General Rare Disease Resources**
https://rarediseases.org/
https://www.eurordis.org/
http://www.agsa-geneticsupport.org.au/

^*^ Working links as of August 2018.

Rare disease patients are dispersed geographically. This makes is difficult for patients to reach expert centers and clinicians who are experienced in using specialized fertility-inducing regimens. Therefore, leveraging technology to reach dispersed patients and connecting patients with specialists is a key part of improving care for CHH/KS. As demonstrated in a recent needs assessment (30), patient partnerships combined with a web-based approach is a highly effective. Patients with rare diseases are internet “power-users” who go online to learn about their condition, access care, and connect with other patients (59). An international network of CHH/KS clinicians and researchers partnered with patients and advocates to respond to the unmet needs identified in the needs assessment. Together, they collaboratively developed a virtual toolkit to help patients learn about their condition, find clinical centers, access genetic testing services and join peer-to-peer support groups (58). This example highlights how respectful and trusting patient-provider partnerships can use co-creation to translate research into improved clinical care. Importantly, for long-term sustainability of such platforms, ongoing patient engagement and participation will be essential.

## Future Directions

There are a number of unanswered questions related to CHH/KS. Studying rare diseases and developing an evidence base to guide best practices is challenging as publications typically come from single centers and have relatively small populations. One opportunity is for broader, international collaboration with harmonized definitions and measures. Patient registries are particularly helpful for rare disease research (60). Such tools can be used to conduct natural history studies and better understand long-term health outcomes and impact on quality of life.

For CHH/KS, such a natural history study could be extremely useful for exploring the phenomenon of reversible CHH (61). Data suggest that ~10% of patients recover function of their hypothalamic-pituitary-gonadal axis and can sustain normal sex steroid levels and fertility following discontinuation of hormonal treatment (62). Interestingly, reversal is not always lasting and these patients appear susceptible to relapse and a subsequent “crash” of their reproductive axis (63). Long-term studies of such cases could potentially help identify biomarkers and predictors for reversal and potentially open new avenues for developing novel treatments. Registries and natural history studies can also help examine how CHH/KS evolves over time and the impact on quality of life. These data can be used to identify patient-reported outcome measures (PROMs) (64, 65). Subsequently, identified PROMs could be used as outcomes and secondary endpoints for clinical trials (66). Indeed, further work is needed to clarify the optimal treatment(s) for CHH/KS and the best timing for treatment initiation (1). Thus, a patient registry and natural history study could help advance the field.

Additional future directions include developing tools and identifying biomarkers to facilitate early diagnosis. Genetic testing can be informative in approximately half of cases. However, an unmet need is access to decisional support for genetic testing. Online discussions and unpublished data indicate that patients may struggle with genetic testing decisions—particularly related to the complex genetics of CHH/KS (5). Patients could benefit from decisional support, interventions promoting active coping strategies and approaches supporting effective family communication of risk. Currently, expertise in specialized fertility-inducing treatment is limited and dispersed. Future directions may include international multicenter trials to determine optimal treatment regimens and using web-based “e-consulting” to share this dispersed, specialized expertise. More work is needed to develop and test effective both face-to-face and web-based interventions for activating and empowering patients for long-term adherence and self-management. Additionally, transitional care has only recently gained attention. As such, there is a limited evidence base for supporting best practices (or exemplar models) for effectively transitioning patients from pediatric to adult oriented care.

## Conclusions

Congenital hypogonadotropic hypogonadism and Kallmann syndrome (CHH/KS) is a rare, treatable form of infertility. Like other rare disease patients, individuals with CHH/KS frequently experience feelings of isolation and alienation. Effective hormonal treatments are readily available for inducing secondary sexual characteristics and fertility (in the vast majority of cases). Indeed, CHH/KS is not life threatening, but it is a severely life-altering condition. Disrupted puberty can have lasting psychological, emotional, and sexual effects. As part of comprehensive care, clinicians should give appropriate attention, care and referrals (e.g., peer-to-peer support, mental health services) as appropriate to ameliorate the psychological aspects of CHH/KS.

## Author Contributions

All authors listed have made a substantial, direct and intellectual contribution to the work, and approved it for publication.

### Conflict of Interest Statement

The authors declare that the research was conducted in the absence of any commercial or financial relationships that could be construed as a potential conflict of interest.

## References

[1] Boehm U Bouloux PM Dattani MT de Roux N Dode C Dunkel L. Expert consensus document: European consensus statement on congenital hypogonadotropic hypogonadism–pathogenesis, diagnosis and treatment. Nat Rev Endocrinol. (2015) 11:547–64. 10.1038/nrendo.2015.112 26194704

[2] Costa-Barbosa FA Balasubramanian R Keefe KW Shaw ND Al-Tassan N Plummer L. Prioritizing genetic testing in patients with Kallmann syndrome using clinical phenotypes. J Clin Endocrinol Metab. (2013) 98:E943–53. 10.1210/jc.2012-4116 23533228 PMC3644607

[3] Young J. Approach to the male patient with congenital hypogonadotropic hypogonadism. J Clin Endocrinol Metab. (2012) 97:707–18. 10.1210/jc.2011-1664 22392951

[4] Stamou MI Cox KH Crowley WF Jr. Discovering genes essential to the hypothalamic regulation of human reproduction using a human disease model: adjusting to life in the “-omics” era. Endocrine Rev. (2016) 2016:4–22. 10.1210/er.2015-1045.2016.1 27454361 PMC6958992

[5] Maione L Dwyer AA Francou B Guiochon-Mantel A Binart N Bouligand J. Genetics in endocrinology: genetic counseling for congenital hypogonadotropic hypogonadism and Kallmann syndrome: new challenges in the era of oligogenism and next-generation sequencing. Euro J Endocrinol. (2018) 178:R55–80. 10.1530/EJE-17-0749 29330225

[6] Palmert MR Dunkel L. Clinical practice. delayed puberty. N Engl J Med. (2012) 366:443–53. 10.1056/NEJMcp1109290 22296078

[7] Harrington J Palmert MR. Clinical review: distinguishing constitutional delay of growth and puberty from isolated hypogonadotropic hypogonadism: critical appraisal of available diagnostic tests. J Clin Endocrinol Metab. (2012) 97:3056–67. 10.1210/jc.2012-1598 22723321

[8] Grumbach MM. A window of opportunity: the diagnosis of gonadotropin deficiency in the male infant. J Clin Endocrinol Metab. (2005) 90:3122–7. 10.1210/jc.2004-2465 15728198

[9] Quinton R Mamoojee Y Jayasena CN Young J Howard S Dunkel L. Society for endocrinology UK guidance on the evaluation of suspected disorders of sexual development: emphasizing the opportunity to predict adolescent pubertal failure through a neonatal diagnosis of absent minipuberty. Clin Endocrinol. (2017) 86:305–6. 10.1111/cen.13257 27749014

[10] Dwyer AA Jayasena CN Quinton R. Congenital hypogonadotropic hypogonadism: implications of absent mini-puberty. Minerva Endocrinol. (2016) 41:188–95. 27213784

[11] Howard SR Dunkel L. Management of hypogonadism from birth to adolescence. Best Pract Res Clin Endocrinol Metab. (2018) 32:355–72. 10.1016/j.beem.2018.05.011 30086863

[12] Klein DA Emerick JE Sylvester JE Vogt KS. Disorders of puberty: an approach to diagnosis and management. Am Fam Physician. (2017) 96:590–9 29094880

[13] Waldstreicher J Seminara SB Jameson JL Geyer A Nachtigall LB Boepple PA. The genetic and clinical heterogeneity of gonadotropin-releasing hormone deficiency in the human. J Clin Endocrinol Metab. (1996) 81:4388–95. 10.1210/jcem.81.12.8954047 8954047

[14] Zhu J Choa RE Guo MH Plummer L Buck C Palmert MR. A shared genetic basis for self-limited delayed puberty and idiopathic hypogonadotropic hypogonadism. J Clin Endocrinol Metab. (2015) 100:E464–54. 10.1210/jc.2015-1080 25636053 PMC4399304

[15] Herman-Giddens ME Slora EJ Wasserman RC Bourdony CJ Bhapkar MV Koch GG. Secondary sexual characteristics and menses in young girls seen in office practice: a study from the pediatric research in office settings network. Pediatrics. (1997) 99:505–12. 10.1542/peds.99.4.505 9093289

[16] Dzemaili S Tiemensma J Quinton R Pitteloud N Morin D Dwyer AA. Beyond hormone replacement: quality of life in women with congenital hypogonadotropic hypogonadism. Endocr Connect. (2017) 6:404–12. 10.1530/EC-17-0095 28698240 PMC5551425

[17] Herman-Giddens ME Steffes J Harris D Slora E Hussey M Dowshen SA. Secondary sexual characteristics in boys: data from the pediatric research in office settings network. Pediatrics. (2012) 130:e1058–68. 10.1542/peds.2011-3291 23085608

[18] Dwyer AA Quinton R Pitteloud N Morin D. Psychosexual development in men with congenital hypogonadotropic hypogonadism on long-term treatment: a mixed methods study. Sex Med. (2015) 3:32–41. 10.1002/sm2.50 25844173 PMC4380912

[19] EURORDIS What is a rare disease? Rare Disease Factsheet. (2007). Available online at: https://www.eurordis.org/sites/default/files/publications/Fact_Sheet_RD.pdf (accessed August, 2018).

[20] Dunkel L Quinton R. Transition in endocrinology: induction of puberty. Euro J Endocrinol. (2014) 170:R229–39. 10.1530/EJE-13-0894 24836550

[21] Nabhan Z Eugster EA. Hormone replacement therapy in children with hypogonadotropic hypogonadism: where do we stand? Endocr Pract. (2013) 19:968–71. 10.4158/EP13101.OR 23807524

[22] Dwyer AA Raivio T Pitteloud N. Gonadotrophin replacement for induction of fertility in hypogonadal men. Best Pract Res Clin Endocrinol Metab. (2015) 29:91–103. 10.1016/j.beem.2014.10.005 25617175

[23] Gronier H Peigne M Catteau-Jonard S Dewailly D Robin G. [Ovulation induction by pulsatile GnRH therapy in 2014: literature review and synthesis of current practice]. Gynecol Obstet Fertil. (2014) 42:732–40. 10.1016/j.gyobfe.2014.07.017 25245838

[24] Li RH Ng EH. Management of anovulatory infertility. Best Pract Res Clin Obstet Gynaecol. (2012) 26:757–68. 10.1016/j.bpobgyn.2012.05.004 22703626

[25] Martin KA Hall JE Adams JM Crowley WF Jr. Comparison of exogenous gonadotropins and pulsatile gonadotropin-releasing hormone for induction of ovulation in hypogonadotropic amenorrhea. J Clin Endocrinol Metab. (1993) 77:125–9. 10.1210/jc.77.1.125 8325934

[26] Martin K Santoro N Hall J Filicori M Wierman M Crowley WF Jr. Clinical review 15: management of ovulatory disorders with pulsatile gonadotropin-releasing hormone. J Clin Endocrinol Metab. (1990) 71:1081A. 10.1210/jcem-71-5-1081 2229271

[27] Pazderska A Mamoojee Y Artham S Miller M Ball SG Cheetham T. Safety and tolerability of one-year intramuscular testosterone regime to induce puberty in older men with CHH. Endocr Connect. (2018) 7:133–8. 10.1530/EC-17-0241 29298845 PMC5754506

[28] Santhakumar A Miller M Quinton R. Pubertal induction in adult males with isolated hypogonadotropic hypogonadism using long-acting intramuscular testosterone undecanoate 1-g depot (Nebido). Clin Endocrinol. (2014) 80:155–7. 10.1111/cen.12160 23383861

[29] Dwyer AA Tiemensma J Quinton R Pitteloud N Morin D. Adherence to treatment in men with hypogonadotrophic hypogonadism. Clin Endocrinol. (2017) 86:377–83. 10.1111/cen.13236 27647266

[30] Dwyer AA Quinton R Morin D Pitteloud N. Identifying the unmet health needs of patients with congenital hypogonadotropic hypogonadism using a web-based needs assessment: implications for online interventions and peer-to-peer support. Orphanet J Rare Dis. (2014) 9:83. 10.1186/1750-1172-9-83 24915927 PMC4059885

[31] Horne R Weinman J. Patients' beliefs about prescribed medicines and their role in adherence to treatment in chronic physical illness. J Psychosom Res. (1999) 47:555–67. 10.1016/S0022-3999(99)00057-4 10661603

[32] Huffer V Scott WH Connor TB Lovice H. Psychological studies of adult male patients with sexual infantilism before and after androgen therapy. Ann Inter Med. (1964) 61:255–68. 10.7326/0003-4819-61-2-255 14208163

[33] Cohen JS Biesecker BB. Quality of life in rare genetic conditions: a systematic review of the literature. Am J Med Genet Part A. (2010) 152A:1136–56. 10.1002/ajmg.a.33380 20425818 PMC3113481

[34] Holtzclaw Williams P. Policy framework for rare disease health disparities. Policy Polit. Nurs. Pract. (2011) 12:114–8. 10.1177/1527154411404243 21486874

[35] Maione L Colao A Young J. Bone mineral density in older patients with never-treated congenital hypogonadotropic hypogonadism. Endocrine. (2018) 59:231–3. 10.1007/s12020-017-1334-1 28577250

[36] Ozbek MN Demirbilek H Baran RT Baran A. Bone mineral density in adolescent girls with hypogonadotropic and hypergonadotropic hypogonadism. J Clin Res Pediat Endocrinol. (2016) 8:163–9. 10.4274/jcrpe.2228 27087454 PMC5096471

[37] Laitinen EM Hero M Vaaralahti K Tommiska J Raivio T. Bone mineral density, body composition and bone turnover in patients with congenital hypogonadotropic hypogonadism. Int J Androl. (2012) 35:534–40. 10.1111/j.1365-2605.2011.01237.x 22248317

[38] Schulz KM Sisk CL. The organizing actions of adolescent gonadal steroid hormones on brain and behavioral development. Neurosci Biobehav Rev. (2016) 70:148–58. 10.1016/j.neubiorev.2016.07.036 27497718 PMC5074860

[39] Hier DB Crowley WF Jr. Spatial ability in androgen-deficient men. N Engl J Med. (1982) 306:1202–5. 10.1056/NEJM198205203062003 7070432

[40] Lasaite L Ceponis J Preiksa RT Zilaitiene B. Impaired emotional state, quality of life and cognitive functions in young hypogonadal men. Andrologia. (2014) 46:1107–12. 10.1111/and.12199 24313565

[41] Lašaitė L Čeponis J Preikša RT Žilaitienė B. Effects of two-year testosterone replacement therapy on cognition, emotions and quality of life in young and middle-aged hypogonadal men. Andrologia. (2017) 49:e12633. 10.1111/and.12633 27545990

[42] Lewkowitz-Shpuntoff HM Hughes VA Plummer L Au MG Doty RL Seminara SB. Olfactory phenotypic spectrum in idiopathic hypogonadotropic hypogonadism: pathophysiological and genetic implications. J Clin Endocrinol Metab. (2012) 97:E136–44. 10.1210/jc.2011-2041 22072740 PMC3251934

[43] Croy I Negoias S Novakova L Landis BN Hummel T. Learning about the functions of the olfactory system from people without a sense of smell. PLoS ONE. (2012) 7:e33365. 10.1371/journal.pone.0033365 22457756 PMC3310072

[44] Kaplowitz PB. Delayed puberty. Pediatr Rev. (2010) 31:189–95. 10.1542/pir.31-5-189 20435710

[45] Golub MS Collman GW Foster PM Kimmel CA Rajpert-De Meyts E Reiter EO. Public health implications of altered puberty timing. Pediatrics. (2008) 121(Suppl 3):S218–30. 10.1542/peds.2007-1813G 18245514

[46] Thapar A Collishaw S Pine DS Thapar AK. Depression in adolescence. Lancet. (2012) 379:1056–67. 10.1016/S0140-6736(11)60871-4 22305766 PMC3488279

[47] Michaud PA Suris JC Deppen A Gender-related psychological and behavioural correlates of pubertal timing in a national sample of Swiss adolescents. Mol Cell Endocrinol. (2006) 254–5:172–8. 10.1016/j.mce.2006.04.037 16806671

[48] Bobrow NA Money J Lewis VG. Delayed puberty, eroticism, and sense of smell: a psychological study of hypogonadotropinism, osmatic and anosmatic (Kallmann's syndrome). Arch Sex Behav. (1971) 1:329–44. 10.1007/BF01638061 24179080

[49] Huisman J Bosch JD Delemarre vd Waal HA. Personality development of adolescents with hypogonadotropic hypogonadism. Psychol Rep. (1996) 79:1123–6. 10.2466/pr0.1996.79.3f.1123 9009757

[50] Bouvattier C Maione L Bouligand J Dode C Guiochon-Mantel A Young J. Neonatal gonadotropin therapy in male congenital hypogonadotropic hypogonadism. Nat Rev Endocrinol. (2012) 8:172–82. 10.1038/nrendo.2011.164 22009162

[51] Aydogan U Aydogdu A Akbulut H Sonmez A Yuksel S Basaran Y. Increased frequency of anxiety, depression, quality of life and sexual life in young hypogonadotropic hypogonadal males and impacts of testosterone replacement therapy on these conditions. Endocr J. (2012) 59:1099–105. 10.1507/endocrj.EJ12-0134 22972022

[52] Varimo T Hero M Laitinen EM Sintonen H Raivio T. Health-related quality of life in male patients with congenital hypogonadotropic hypogonadism. Clin Endocrinol (Oxf). (2015) 83:141–3. 10.1111/cen.12701 25515567

[53] Shiraishi K Oka S Matsuyama H. Assessment of quality of life during gonadotrophin treatment for male hypogonadotrophic hypogonadism. Clin Endocrinol. (2014) 81:259–65. 10.1111/cen.12435 24612103

[54] Dwyer AA Quinton R. The metabolic syndrome in central hypogonadotrophic hypogonadism. Front Hormone Res. (2018) 49:156–69. 10.1159/000485998 29895006

[55] Picker Institute's Eight Principles of Patient-Centered Care (2015). Available online at: https://nexusipe.org/informing/resource-center/picker-institute%E2%80%99s-eight-principles-patient-centered-care. (accessed August, 2018).

[56] Dwyer AA Pitteloud N. Transition of care from childhood to adulthood: congenital hypogonadotropic hypogonadism. Endocrine Develop. (2018) 33:82–98. 10.1159/000487527 29886503

[57] Dwyer AA Phan-Hug F Hauschild M Elowe-Gruau E Pitteloud N. Transition in endocrinology: hypogonadism in adolescence. Euro J Endocrinol. (2015) 173:R15–24. 10.1530/EJE-14-0947 25653257

[58] BM CA Badiu C Bonomi M Borshchevsky I Cools M Craen M Developing and evaluating rare disease educational materials co-created by expert clinicians and patients: the paradigm of congenital hypogonadotropic hypogonadism. Orphanet J Rare Dis. (2017) 12:57 10.1186/s13023-017-0608-2 28320476 PMC5359990

[59] Fox S Peer-to-Peer Healthcare: Many People - Especially Those Living With Chronic or Rare Diseases - Use Online Connections to Supplement Professional Medical Advice. Washington, DC: Pew Internet, Pew Research Center (2011).

[60] Dasenbrook EC Sawicki GS. Cystic fibrosis patient registries: a valuable source for clinical research. J Cyst Fibros. (2018) 17:433–40. 10.1016/j.jcf.2018.03.001 29555479

[61] Dwyer AA Raivio T Pitteloud N. Management of endocrine disease: reversible hypogonadotropic hypogonadism. Euro J Endocrinol. (2016) 174:R267–74. 10.1530/EJE-15-1033 26792935

[62] Raivio T Falardeau J Dwyer A Quinton R Hayes FJ Hughes VA. Reversal of idiopathic hypogonadotropic hypogonadism. N Engl J Med. (2007) 357:863–73. 10.1056/NEJMoa066494 17761590

[63] Sidhoum VF Chan YM Lippincott MF Balasubramanian R Quinton R Plummer L. Reversal and relapse of hypogonadotropic hypogonadism: resilience and fragility of the reproductive neuroendocrine system. J Clin Endocrinol Metab. (2014) 99:861–70. 10.1210/jc.2013-2809 24423288 PMC3942233

[64] Slade A Isa F Kyte D Pankhurst T Kerecuk L Ferguson J. Patient reported outcome measures in rare diseases: a narrative review. Orphanet J Rare Dis. (2018) 13:61. 10.1186/s13023-018-0810-x 29688860 PMC5914068

[65] Morel T Cano SJ. Measuring what matters to rare disease patients - reflections on the work by the IRDiRC taskforce on patient-centered outcome measures. Orphanet J Rare Dis. (2017) 12:171. 10.1186/s13023-017-0718-x 29096663 PMC5667521

[66] Benjamin K Vernon MK Patrick DL Perfetto E Nestler-Parr S Burke L. Patient-reported outcome and observer-reported outcome assessment in rare disease clinical trials: an ISPOR COA emerging good practices task force report. Value Health. (2017) 20:838–55. 10.1016/j.jval.2017.05.015 28712612

